# NAVIGATOR: an Italian regional imaging biobank to promote precision medicine for oncologic patients

**DOI:** 10.1186/s41747-022-00306-9

**Published:** 2022-11-08

**Authors:** Rita Borgheresi, Andrea Barucci, Sara Colantonio, Gayane Aghakhanyan, Massimiliano Assante, Elena Bertelli, Emanuele Carlini, Roberto Carpi, Claudia Caudai, Diletta Cavallero, Dania Cioni, Roberto Cirillo, Valentina Colcelli, Andrea Dell’Amico, Domnico Di Gangi, Paola Anna Erba, Lorenzo Faggioni, Zeno Falaschi, Michela Gabelloni, Rosa Gini, Lucio Lelii, Pietro Liò, Antonio Lorito, Silvia Lucarini, Paolo Manghi, Francesco Mangiacrapa, Chiara Marzi, Maria Antonietta Mazzei, Laura Mercatelli, Antonella Mirabile, Francesco Mungai, Vittorio Miele, Maristella Olmastroni, Pasquale Pagano, Fabiola Paiar, Giancarlo Panichi, Maria Antonietta Pascali, Filippo Pasquinelli, Jorge Eduardo Shortrede, Lorenzo Tumminello, Luca Volterrani, Emanuele Neri, on behalf of the NAVIGATOR Consortium Group

**Affiliations:** 1 Academic Radiology, Department of Translational Research and of New Surgical and Medical Technology, University of Pisa, 56126 Pisa, Italy; 2 “Nello Carrara” Institute of Applied Physics — IFAC of the National Research Council — CNR, Florence, Italy; 3 Institute of Information Science and Technologies — ISTI of the National Research Council — CNR, Pisa, Italy; 4 Department of Radiology, Careggi University Hospital, Florence, Italy; 5 Azienda USL Toscana Centro, Florence, Italy; 6 Azienda Regionale di Sanità, Florence, Italy; 7 Department of Computer Science and Technology of the University of Cambridge, Cambridge, UK; 8 Unit of Diagnostic Imaging, Department of Medical, Surgical and Neurosciences and of Radiological Sciences, University of Siena, Azienda Ospedaliero-Universitaria Senese, Siena, Italy; 9 Italian Society of Medical and Interventional Radiology, SIRM Foundation, Milan, Italy

**Keywords:** Artificial intelligence, Biobanks, Biomarkers, Precision medicine, Radiomics

## Abstract

NAVIGATOR is an Italian regional project boosting precision medicine in oncology with the aim of making it more predictive, preventive, and personalised by advancing translational research based on quantitative imaging and integrative omics analyses. The project’s goal is to develop an open imaging biobank for the collection and preservation of a large amount of standardised imaging multimodal datasets, including computed tomography, magnetic resonance imaging, and positron emission tomography data, together with the corresponding patient-related and omics-related relevant information extracted from regional healthcare services using an adapted privacy-preserving model. The project is based on an open-source imaging biobank and an open-science oriented virtual research environment (VRE). Available integrative omics and multi-imaging data of three use cases (prostate cancer, rectal cancer, and gastric cancer) will be collected. All data confined in NAVIGATOR (*i.e.,* standard and novel imaging biomarkers, non-imaging data, health agency data) will be used to create a *digital patient model*, to support the reliable prediction of the disease phenotype and risk stratification. The VRE that relies on a well-established infrastructure, called *D4Science.org*, will further provide a multiset infrastructure for processing the integrative omics data, extracting specific radiomic signatures, and for identification and testing of novel imaging biomarkers through big data analytics and artificial intelligence.

## Key points

- NAVIGATOR aims to support precision medicine in oncological patients.
- NAVIGATOR biobank delivers an infrastructure for integrative omics and multi-imaging data.
- NAVIGATOR incorporates a digital patient model ensuring accurate cancer phenotyping.
- NAVIGATOR biobank offers a virtual research environment to extract radiomics-based biomarkers.

## Background

Biobanks are born as repositories of biological materials and related data, which are collected for a broad spectrum of further and future analysis (*e.g.,* genetic, genomic, epigenetic, miRNA, proteomics, and transcriptomics) [1]. Only in the last few years, they have also begun to include imaging data, and real imaging biobanks have begun to exist.

The Imaging Biobanks Working Group of the Research Committee was established by the European Society of Radiology in 2014 [2]. They defined imaging biobanks as ‘organized databases of medical images, and associated imaging biomarkers shared among multiple researchers, linked to other biorepositories’. From this definition, it is evident that an imaging biobank is not simply a system of archiving and transmitting images as are the picture archiving and communication systems (PACS) used in hospitals. An imaging biobank not only allows the storage and retrieval of medical images and associated metadata, but the added value is that these data are linked to the imaging biomarkers, extracted through the radiomics analysis of the imaging data (typically radiology imaging data) and to clinical, molecular, biological, and genomic data of a typical tissue biobank. The big potential of imaging biobanks is the ability to provide integrative omics data for studying advanced imaging techniques on imaging pools with sufficient sample size. This availability of data is necessary for the researchers to find an association between phenotype and genotype [3], to design and validate new imaging biomarkers, and to understand their biological significance, which may be a crucial point in precision medicine [4]. Once large high-quality and well-curated data sets are available within the biobank, they can be used for data mining.

In this article, we describe an establishment of Italian regional imaging biobank project, NAVIGATOR, its infrastructure, ethical and sustainability challenges, research and clinical consortium structure, the future development, and prospective.

## A regional imaging biobank

NAVIGATOR is an Italian regional project that is designed to develop an imaging biobank open to various stakeholder communities and devoted initially to the oncological domain of solid tumours. The main deliverables and milestones of the project are summarised in Table 1.

**Table 1:** The main deliverables and milestones of the NAVIGATOR project

Deliverable number	Deliverable description	Milestone number	Milestone description
D1.1	Assessment of the project activities	MIL1	Startup of the activities for project management
D1.2	Report on innovation and risk management		
D1.3	Report on awareness and dissemination plan		
D1.4	Public website		
D2.1	NAVIGATOR data model		No milestones expected
D2.2	NAVIGATOR D4Science services functional specification: biobank and VREs		
D3.1	Report on NAVIGATOR biobank content	MIL5 MIL6	D4Science services in support of NAVIGATOR VREs D4Science services in support of NAVIGATOR biobank and aggregation of data sources
D3.2	Report on NAVIGATOR biobank biomarkers	MIL7 MIL8	NAVIGATOR biobank with data collected from hospitals and Agenzia Regionale di Sanità NAVIGATOR biobank with algorithms for biomarkers
D4.1	Preliminary results about image and data acquisition and segmentation	MIL2	Establishment of standardised acquisition and segmentation protocols and reports
D4.2	Report on data acquisition and curation for the three clinical use cases		
D5.1	Gold standard and radiomics biomarkers for prostate, gastric colorectal cancer	MIL3	Definition of ‘gold-standard’ biomarkers, radiomics biomarkers, and data analytics and artificial intelligence tools that will be implemented in the VRE
D5.2	Report on image analysis using radiomics and ‘gold-standard’ imaging biomarkers		
D5.3	Report on artificial intelligence-based data analysis		
D6.1	Report on data privacy and security policies: anonymisation and GDPR	MIL4	Acquisition of the definition of patient’s personal data associated with clinical images
D6.2	Report on the prior informed consent of NAVIGATOR biobank		
D6.3	Report on the reutilisation of medical imaging and personal data acquired before the project		
D6.4	NAVIGATOR joint controlled agreement		
D6.5	Material transfer agreement		
D6.6	Report on intellectual property rights in aggregated data		
D6.7	Biobank access regulation		
D6.8	Market studies for biobank services		
D6.9	State of biobank governance		

*GDPR* General Data Protection Regulation, *VRE* Virtual research environment

In Europe, several projects aim to build an imaging biobank, such as the euCanSHare project [5], the PRIMAGE project [6], or the Lifebrain project [7], but most of them are under development as part of SC1-DT-TDS-05-2020 H2020 call ‘AI for Health Imaging’ [8]. The creation of an H2020 call entirely dedicated to the development of large cancer imaging repositories demonstrates that Europe has underlined the importance of access to multicentre cancer imaging datasets for the artificial intelligence (AI) communities and industries.

Despite this great international interest, in Italy, there are currently no examples of imaging biobanks; therefore, the creation of an oncologic imaging biobank in Tuscany is a challenge that could help the development of precision medicine in oncology in Italy. Although NAVIGATOR differs from PACS in several aspects, it will follow the work and future developments of the *regional PACS* initiative, to promptly deliver strategies and solutions for the smooth integration of the biobank with this system. This will further enable the positioning of NAVIGATOR as an additional service of the regional healthcare system. NAVIGATOR will deliver proper image acquisition guidelines and ethical, security, and privacy operation policies (*i.e.,* governance) that may be needed to establish the biobank as a regional service. Then, it will be possible to connect NAVIGATOR to the other existing biobank infrastructures such as the European Biobanking and BioMolecular resources Research Infrastructure-European Research Infrastructure Consortium [9].

Nowadays, some imaging biomarkers are routinely used to support decision-making in cancer clinical management [10–15]. However, the integration of imaging biomarkers into clinical practice is still restricted to a limited number of imaging biomarkers due to several shortcomings: harmonisation of data acquisition and analysis, lack of international standards, and availability of good quality validated data sets. To overcome these criticalities, the development of imaging biobanks is an emerging field proposed as the radiological counterpart of the more common biobank of biological materials. The NAVIGATOR project inserts into this context aiming to the realisation of a regional imaging biobank.

Since the beginning of this last decade, radiomics has represented the first real attempt to transform the old qualitative or semiquantitative clinical imaging into the complete data-driven and quantitative perspective, trying to embrace the emerging paradigm of the science of big data and AI [16–21]. Specifically, since the NAVIGATOR project was created as an imaging biobank, the main focus will be the analysis of the information extracted from these images, using radiomics and conventional machine learning on the one hand and images and deep learning directly on the other. From a methodological point of view, we will focus on reproducibility, accountability, explainability, and interpretability of our machine learning models to obtain valuable and safe results. An example of the typical analysis workflow (radiomic feature extraction associated with conventional machine learning and deep learning processing) performed on a cohort of prostate cancer patients participating in the NAVIGATOR project can be found in the paper by Bertelli et al. [22].

The framework offered by NAVIGATOR has the key ingredients to get improvements in AI and radiomics applied to medical imaging: researchers with diverse backgrounds (clinicians, radiologists, biomedical engineers, information scientists, physicists, mathematicians) will join their efforts to collect large and curated data (following acquisition standards) and to design, develop, and perform processing pipelines based on deep learning and radiomics to provide the community with data and innovative tools for the automatic analysis of radiological data and metadata.

## Ethical and legal challenges

Among the members of the European Union (EU), only a few legal systems have adopted a special law on research biobanks, establishing organisational requirements for the infrastructure and the management of biological samples, as well as the protection of the rights of the subjects involved, which must be reconciled with the needs of science. In the Italian legal system, which is characterised as a hybrid system [23], the discipline of the establishment of biobanks is placed in the face of the spaces left empty by the law. This discipline is often the result of the interpretations of individuals and soft law instruments operating on principles that are often formed in the practice of those individuals operating in the sector.

Ultimately, it all depends on the absence of a law aimed at governing the phenomenon in an orderly manner. In the absence of a specific binding regulatory framework, the approach becomes how to properly use the existing regulation with other sectors. We appeal to the rules governing the protection of personal data and the provisions governing clinical trials, always making use of the classic categories of the Italian civil code. These schemes have allowed operators to trace a legal horizon; however, they do not always provide an adequate regime. Each model borrowed, albeit for different reasons, could be deficient and inappropriate. Even the EU Clinical Trials Regulation [24] is not a model that follows specific challenges and needs of research biobanks, primarily because the establishment of biobanks does not imply any clinical trials; moreover, the provisions on experimentation do not contemplate the hypothesis of a ‘sharing of the material’ differently from what happens in research [25]. The legislator will soon no longer be able to exempt itself from dealing with research biobanks as a research model in which different skills — ethics, information technology, medicine, law, and aspects of the administration — are called into question.

In the lack of specific regulation for the biobank phenomenon, the experience of others becomes precious. In this framework, the experience of the project NAVIGATOR could be analysed and taken into consideration.

## Partnership

For the successful design and implementation of NAVIGATOR, a very high level of interdisciplinary consortium was required, with expertise ranging from protocols for imaging acquisition and reporting to AI model development. The NAVIGATOR consortium relies on a strong regional network of hospitals, university hospitals, and research institutions, whose multidisciplinary expertise guarantees coverage of all the requirements.

On the clinical side, the NAVIGATOR consortium has the possibility of reuniting the three territorial areas of the public regional health system. This represents a strength of the project, which can therefore include a wide range of cases and therefore greater reliability and effectiveness in objective research. The University of Pisa (UNIPI), the Azienda USL Toscana Centro (AUSL TC), the Azienda Ospedaliera Universitaria Senese (AOUS), and the Azienda Ospedaliera Universitaria Careggi (AOUC) constitute the clinical team.

On the technical side, the knowledge and expertise essential for the successful development of NAVIGATOR are given by the research centres’ partners which include the Institute of Information Science and Technologies of the National Research Partner Council (ISTI-CNR) and ‘Nello Carrara’ Institute of Applied Physics of the Partner National Research Council (IFAC-CNR). The Azienda Regionale di Sanità (ARS) will contribute with clinical records and data science expertise. The international actors partnered in NAVIGATOR will support the coordination with other emerging initiatives at the EU level, granting the international bases and timely outreach of project outcomes.

## Ethical and legal aspects of a biobank: moving in the new world data sharing economy

Many ethical and legal issues, which focus on the balance between research activity and the rights and freedoms of individuals, are related to the institution of a research biobank. There is a lack of systematic international or national rules that address these issues. Therefore, this paragraph aims to present a reflection on some ethical-legal aspects addressed in the creation of the NAVIGATOR biobank. This means that the relationship between General Data Protection Regulation (EU) 2016/679 (GDPR) and images must first be understood. According to the Recital n. 51 to the GDPR, the processing of images should not systematically be the processing of special categories of personal data. Clinical images are covered by the definition of biometric data only when processed through a specific technical means that allows the unique identification or authentication of a natural person. In the case of a biobank for medical imaging, the images of clinical examinations do not qualify as personal data unless they can be associated with the personal data of the patient, even if they are pseudonymised. Biobanks for medical imaging will anonymise or pseudonymise the personal data of the patient. For this purpose, a list of personal data of the patient that can be stored and associated with the clinical images for a biobank has been realised at the starting point of the project. The list supports the selection of old clinical images and personal data associated with patients that were acquired before the establishment of the biobank for medical imaging when former patients have not given specific informed consent for biobanking activities. The list of personal data of the patient that can be stored and associated with the clinical images has an impact on several matters for the future biobank: the joint controller agreement for managing health data in the project consortium, the secondary use of associated medical imaging and personal data acquired before the constitution of the biobank, and the consent for the biobanking purpose.

### Joint controller agreement for managing personal health data under GDPR

The issue of sharing personal data in large research consortiums or biobank infrastructures commonly arises and similar cases in which data processing takes place in an intragroup context [26]. According to the aims of the GDPR, organisations are obliged to demonstrate that their processing activities are compliant with the Data Protection Principles (Rec. 85; Art. 5 (2) and Rec. 74; Art. 24 GDPR). An arrangement between joint controllers can help organisations to demonstrate compliance with all the principles of the regulation: principles of lawfulness, fairness and transparency, purpose limitation, data minimisation, accuracy, storage limitation, integrity, and confidentiality. Under the NAVIGATOR project, more controllers jointly determine the purposes and means of the processing of personal data, and they are thus joint controllers (Rec. 79; Art. 4 (7) and Art. 26). The essence of the arrangement shall be made available to the data subject, and special attention will be posted on NAVIGATOR service infrastructure to optimise the value of imaging data in connection with other clinical data (clinical health records). The service infrastructure in the NAVIGATOR project is a platform to manage health personal data. The Guidelines 07/2020 on the concepts of controller and processor in the GDPR, adopted on September 02, 2020, by the European Data Protection Board, remind us that research projects by several research institutes can decide to participate in a specific joint research project and to use to that end the existing platform of one of the institutes involved in the project. Each institute feeds personal data it holds into the platform for joint research and uses the data provided by others through the platform for carrying out the research. In this case, all institutes qualify as joint controllers for the personal data processing that is done by storing and disclosing information from this platform since they have decided together the purpose of the processing and the means to be used (the existing platform). Each of the institutes however is a separate controller for any other processing that may be carried out outside the platform for their respective purposes. A joint controller agreement among the NAVIGATOR partners will be arranged with the elements established by Art. 26 GDPR and will reflect the respective roles and relationships of the joint controllers vis-à-vis the data subjects as well as considering the service infrastructure.

### Consent procedures for collecting medical images

The emergence of biobanks as a vital research tool in the medical sciences has led to a widespread debate in the literature about how to best handle the consent procedures governing the enrolment of participants in research and the subsequent use of participant samples and data in other studies.

Informed consent is an extremely important tool to implement various international, EU, and national mandatory laws (*e.g.,* on the protection of personal data, use of biological material, clinical trials) and, above all, to make biomedical activities consistent with fundamental ethical principles, such as dignity and self-determination (see, *e.g.,* Charter of Fundamental Rights of the European Union, European Convention on Human Rights, Convention on Human Rights and Biomedicine — Oviedo, 4 April 1997, and its Additional Protocols).

To comply with these principles and laws, information must be provided in a way that meets the requirements of the different legal sources in a text that addresses the different topics. Therefore, not only the requirements of a specific legal framework, even as extremely important as Regulation (EU) 2016/679, should be considered but the entire legal and ethical framework underlying informed consent. In any case, Regulation (EU) 2016/679 on the protection of individuals regarding the processing of personal data and on the free movement of such data (hereinafter GDPR) lays down special rules for consent and rights in the context of research activities. When the broad consent model is applied for biobanking activities, general consent is gathered at the time of enrolment. Subsequently, samples stored in the biobank can be used for new studies that fall within the scope of the consent without reobtaining consent from participants. Medical researchers defend the broad model by arguing that it is the best way to make large-scale biobank research feasible.

The GDPR considers the situation in which it is not possible to fully identify the purposes of personal data processing for scientific research at the time of data collection. The derogation of the principle of the ‘granularity’ in research is allowed by the ‘Recital’ no. 33 of the GDPR; the GDPR and other European sources extend the effectiveness of consent. The principle of the limitation of purpose prescribes that ‘the processing of personal data for purposes other than those for which the personal data were initially collected should be allowed only where the processing is compatible with the purposes for which the personal data were initially collected’ (Recital 50), but nevertheless, ‘further processing for archiving purposes in the public interest, scientific or historical research purposes or statistical purposes shall, in accordance with Article 89 (1), not be considered to be incompatible with the initial purposes’ (Article 5 (1) (b) GDPR). For purposes of this type, a sort of presumed consent is given. The same approach was chosen by the Council of Europe in its Recommendation on the Protection of Health-Related Data of 2019 which replaced the above-mentioned Recommendation of 1997 (see Article 4 (1) (b)). This recommendation also seems to consider that it may be difficult to provide detailed information to the data subject about the use of health-related data at the time of collection (see Article 11 (2)). Starting from the aforementioned framework, dealing with the problem of future research depends on the current state of knowledge.

The NAVIGATOR consent form will be realised according to the Recital n. 33, and so, data subjects shall ‘have the opportunity to give their consent only to certain areas of research or parts of research projects to the extent allowed by the intended purpose’ if — and only if — the research project is ‘in keeping with recognised ethical standards for scientific research’. Despite this, such consent should still be in line with the applicable ethical standards for scientific research. Taking into consideration the last affirmation and the meaning of scientific research in the EU framework, we can identify the possible problems in dealing with future research. In relation to biobanking activities and building a research database, the future research has lawful broad consent for the use and reuse of personal data only if further activity could qualify as a ‘genuine research project’ developed in the framework of the activities of public or private research organisations. This is true if the future research is set up by relevant sector-related methodological and ethical standards in conformity with good practice and with the proper oversight of an ethics committee.

## Use cases

The NAVIGATOR clinical team (University of Pisa, Azienda USL Toscana Centro, Azienda Ospedaliera Universitaria Senese, Azienda Ospedaliera Universitaria Careggi) will guarantee the clinical case study for the biobank. Three major abdominal neoplasms have been chosen: prostate cancer, rectal cancer, and gastric cancer.

Prostate cancer is the most prevalent male malignancy [27]. Treatment recommendations are currently based on three levels of risk stratification (low, intermediate, and high) according to prostate-specific antigen, Gleason score, and T category, with active surveillance offered to low-risk men [28]. However, some low-risk patients may harbour diseases that are more aggressive; thus, risk stratification needs to be improved. Magnetic resonance imaging (MRI) plays a crucial role in imaging prostate cancer [29].

Colorectal cancer is the third most common cancer and the third leading cause of cancer deaths in both males and females [30]. The interest for this neoplasm is related to the identification of the locally advanced cancer (T3 stage) with a complete response to neoadjuvant chemoradiotherapy that could be excluded from surgery [31]. The identification of such patients prior to treatment and the response to treatment are based only on MRI imaging [32].

Gastric cancer is the fourth most common neoplasm in the world and the third leading cause of cancer death [33]. The treatment is complex and multimodal today, and the entire decision-making process is largely driven by imaging [34]. The task of imaging is to be able to discern between early and advanced forms of gastric cancer, with the possibility of endoscopic *versus* surgical resections and/or neo-adjuvant therapy, as well as recognise forms liable to multimodal treatment. The prognostic value of microsatellite instability in intestinal-type non-cardia cancer and the different clinical impact of gastric cancers are interesting goals to predict in the pretreatment phase [35]. These neoplasms are divided into mesenchymal type and epithelial type, based on molecular characteristics [35].

The reason for the choice of these three neoplasms was slightly different: for prostate cancer, to improve risk stratification; for rectal cancer, to identify with certainty the complete response to neoadjuvant treatment in the case of stage T3; and for gastric cancer, to identify those forms liable to multimodal treatment and in particular the ones which respond to neoadjuvant therapy and, thus, may benefit from aggressive multimodal treatment. The radiomics-based biomarkers may be an additional tool to better elucidate these questions.

## Platform architecture

**Fig. 1:**
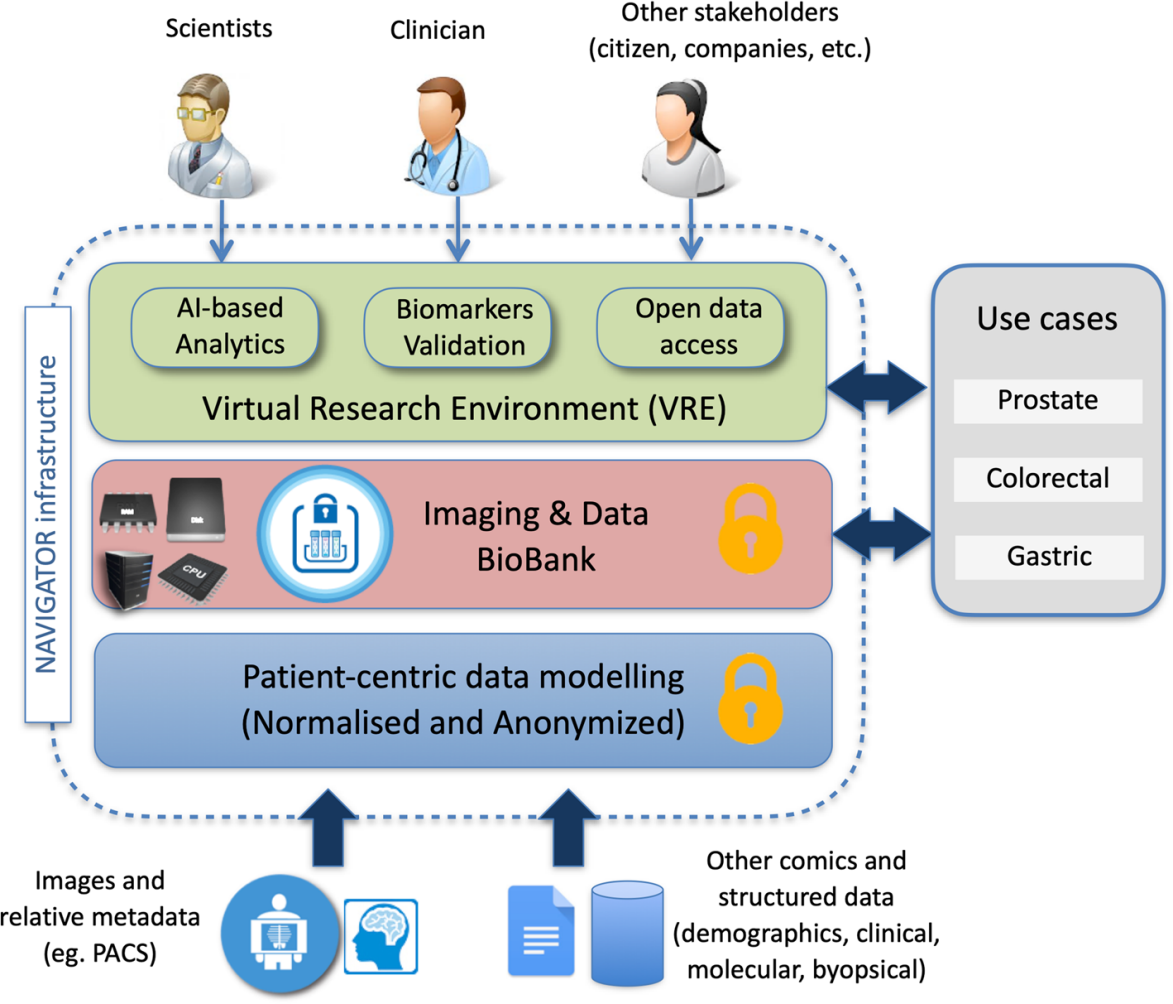
The envisioned architecture of the NAVIGATOR project

The ultimate objective of NAVIGATOR is to deliver the technological platform whose high-level architecture, depicted in Fig. 1, will provide the following:

- An *innovative open imaging biobank* was heterogeneous, anonymised (or pseudonymised) imaging, and related patient data will be collected from multiple clinical sources and shared among multiple researchers, by relying on a flexible data model complaint to state-of-the-art clinical data and metadata standards.
- A *virtual research environment* (VRE) that will offer web access to a digital laboratory where clinicians, scientists, and clinical stakeholders can experiment, discover, test, and validate novel or known biomarkers over the biobank, via a set of available data analytics tools based on radiomics and AI algorithms (Fig. 1).

### The biobank

The NAVIGATOR imaging biobank will integrate heterogeneous, pseudonymised patient-related data from multiple clinical sources, ranging from PACS archives, relative medical reports, and biomarkers to public clinical-health records collected from the database of the Agenzia Regionale di Sanità (Toscana, Italy). The anonymisation tools integrated into the clinical PACS will serve the deletion of sensitive information (*i.e.,* patient’s name and date of birth), which will be maintained by the clinical centres according to the definition of GDPR Recital 26.

The underlying patient centric, agnostic data model will be shaped up by well-established metadata standards — such as Digital Imaging and Communications in Medicine (DICOM), Minimum Information About BIobank data Sharing (MIABIS), Observational Medical Outcomes Partnership (OMOP)-common Data Model (DM) — and devised to flexibly adapt to any form of patient-related data that may be identified and integrated into the biobank in the future. This will ensure the biobank scalability and ability to integrate with tumour types beyond the ones targeted by the project. An initial stage of the project will identify all the clinical and molecular variables to be stored and linked to the imaging data. These variables will be suitably stored in a database implementing an electronic clinical reporting form, while the imaging data will be stored in a dedicated repository of DICOM files, as better detailed in the following.

### The NAVIGATOR VRE

The NAVIGATOR VRE will offer web access to a digital laboratory’ where clinicians, scientists, and clinical stakeholders can upload data into the biobank and collaborate, experiment, discover, test, and validate novel or known biomarkers, thanks to available data analytics tools based on radiomics and AI libraries. VRE will supply the users with the following:

- User dashboard: users can access a local space where they can keep and share data via an online, access-controlled file system (dropbox like); they can integrate, test, and share their methods with others and exchange messages via social tools and mailing lists.
- Experiment handling: users can integrate methods to perform experimental actions in this domain, such as image normalisation, segmentation, feature extraction, classification, and regression; can select existing methods, configure, and experiment with the biobank data or manually uploaded images; and can share their methods and data for the sake of science reproducibility.
- Biobank management: users can upload studies and related images, as well as anonymised patient and diagnostic data, into the biobank.

The VRE will implement a framework envisioned by many in the domain [36, 37], enabling scientific research communities to mine the rich content of multimedia health data and to carry out various types of proofs and validation of imaging biomarkers in a controlled and collaboration-based pipeline environment. Following open-science principles, NAVIGATOR will sustain cooperative research to ensure reproducibility and verifiability of biomarker models, thus fostering standardisation while reducing variability.

## Platform implementation

The NAVIGATOR VRE will be delivered as an extension and customisation of the D4Science.org infrastructure, a well-established production-ready technology today supporting the operation of various European H2020 and Horizon Europe Research Infrastructures (*e.g.,* SoBigData.eu [38], AriadnePlus [https://ariadne-infrastructure.eu/], BlueCloud [https://blue-cloud.org/], AGINFRA [https://plus.aginfra.eu/]). This infrastructure is in turn powered by the gCube software toolkit^1^. *VREs*, *science gateways*, *virtual laboratories*, and other similar terms [39] are used to indicate web-based systems emerged to provide researchers with integrated and user-friendly (transparent) access to data, services, and computing resources of interest for a given investigation that is usually spread across many and diverse data and computing infrastructures. VREs hide to scientists, often without any information technology background, the complexity of sophisticated computing stacks and provide them with intuitive user interfaces they can use to perform experiments, enact collaboration among colleagues, and control access to their algorithms and data, for the sake of investigations.

Many frameworks can be used to build such systems. Shahand et al. [40] have identified eleven frameworks explicitly exploited to develop science gateways including Apache Airavata [https://airavata.apache.org/], Catania SG Gateway [41], Globus [42], HUBzero(+Pegasus) [43], ICAT Job Portal [44], WS-PGRADE/gUSE [45]. The D4Science infrastructure is singles out for its innovative approach offering components and tools to operate a data service infrastructure capable of supporting the dynamic creation of custom VREs by integrating data, algorithms, and services from specific disciplines. Its VREs, available at D4science.org via dedicated portals, are today serving different disciplinary domains [46–49], which (i) include services that operate on the same cloud storage and computation resources, located at Institute of Information Science and Technologies of the National Research Partner Council and partly at GARR Consortium,^2^ and (ii) can rely on state-of-the-art big data analysis tools built on such cloud resources. Overall, D4Science is currently serving more than thousands of users (more than 7,000 in September 2018). In the period January-September 2018, the users served by this infrastructure and its VREs performed: a total of 50,127 sessions, with an average of about 5,569 sessions per month; a total of 4,288 social interactions, with an average of about 476 interactions per month; and a total of 150 million of analytics tasks, with an average of about 16 million tasks per month. The experiences made while exploiting gCube to operate the D4Science.org infrastructure demonstrate not only the production readiness and flexibility of customisation of the software but also that the principles governing VREs delivery and system openness are key in the modern science settings [50].

By realising the NAVIGATOR infrastructure on top of D4Science, we will, therefore, (i) ensure the ability of the biobank and VREs to be sustained over time, (ii) deliver all the flexibility required to extend VRE functionality and biobank features in the future, and (iii) operate a production system by adopting an economy of scale, scalable, and performing approach.

**Fig. 2:**
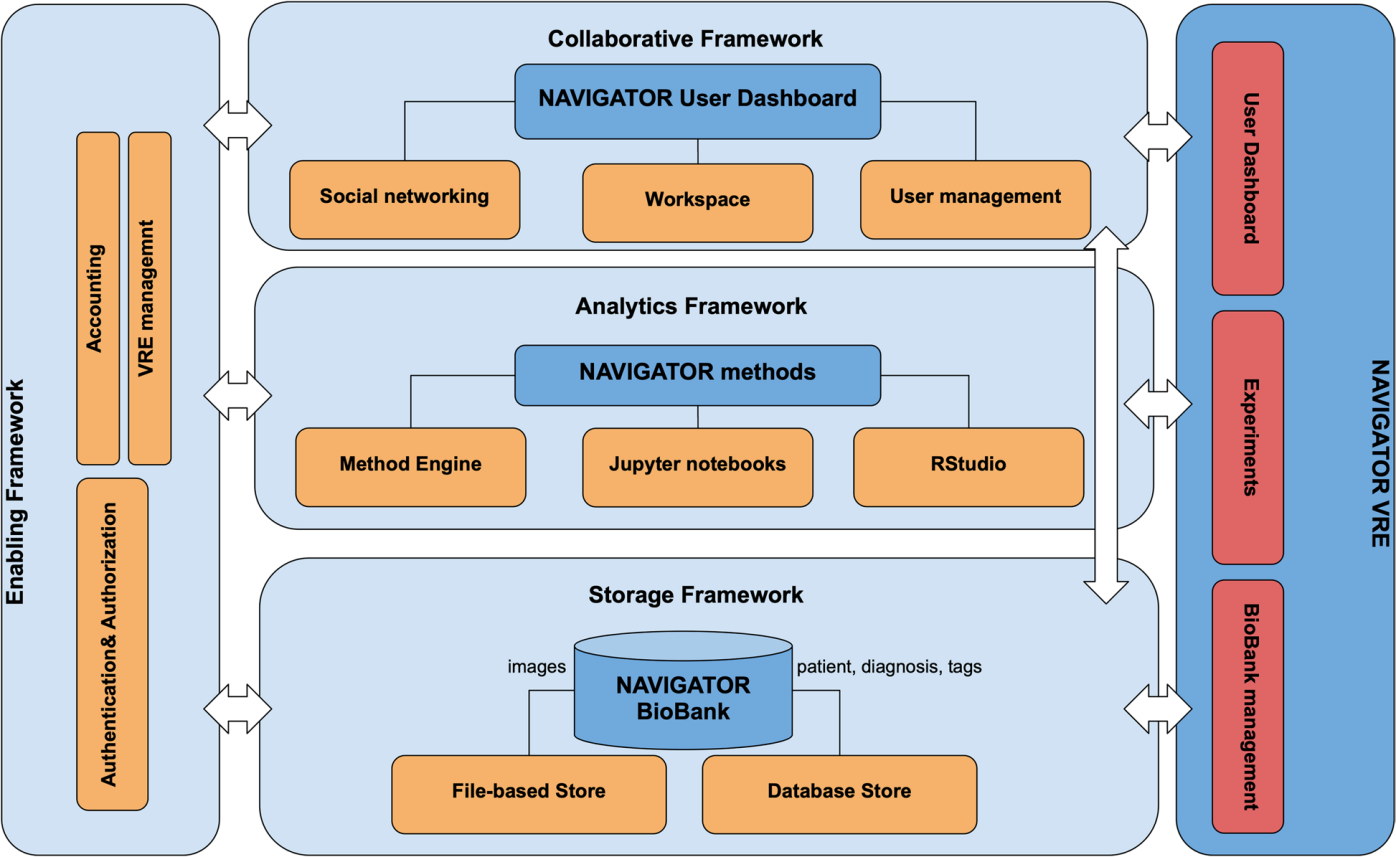
NAVIGATOR virtual research environment (VRE) instantiated over the D4Science platform

As shown in Fig. 2, D4Science operates a three-tier architecture whose components (orange boxes) can be customised to different use cases and applications and be delivered as web applications, via dedicated VREs, to specific groups of users. More specifically, D4Science will be instantiated to deliver the NAVIGATOR biobank and applications as explained in following paragraphs.

### Biobank

The storage framework of D4Science offers a distributed file system accessible to users (humans and services) and a database overlay, supporting several data management systems as a service; examples are SQL databases [https://en.wikipedia.org/wiki/SQL], MongoDBs [https://www.mongodb.com/], TimeSeries DBs [https://en.wikipedia.org/wiki/Time_series_database], Graph Stores [https://en.wikipedia.org/wiki/Graph_database], etc. The biobank will be built as a dedicated Postgres [https://www.postgresql.org/] database for the clinical and molecular variables (*i.e.,* Database Store in Fig. 2), in combination with a structured repository of DICOM files for the storage of the related images (*i.e.,* file-based store in Fig.2). The image upload phase ensures that DICOM tags are extracted from the images and inserted into the database to enable query and data filtering based on these.

### VRE

It is instantiated to provide the three sets of functionalities described in the architecture section (red boxes). The web applications will be developed by the project to address the specific requirements of the NAVIGATOR community, ensuring different user typologies are granted access to the application and data with dedicated access rights. The Collaborative Framework offers tools to exchange messages and experiences as well as a workspace (personal file system) where data can be uploaded and shared via fine-grained access control with other users. The analytics framework offers Jupyter notebooks and RStudio environments that users can adopt out of the box to operate over data extracted from the biobank (or otherwise uploaded by the user in the workspace); in scope, the method engine allows scientists to integrate their custom data analytics methods (Java, Python, R, C++, etc.) and web services to share them with other users (individuals or groups) as a service.

## Expected results

### Precision and personalised medicine

Medicine is experiencing a major change in the last decades, moving from reactive to proactive approaches by providing personalised medical solutions. As far as oncology is concerned, precision medicine highly relies on increasingly detailed characterisation of disease states using the multiple omics platforms for better individualise diagnostics, prognostics, and therapeutics, thus setting a cornerstone of integrative omics medicine, the last frontier of medical sciences. Precision medicine holds promise for better personalisation of oncological care; meantime, the personalisation strongly relies on modelling individual case characteristics and their variability, as opposed to the classical approach, named ‘one size fits all’, based on generalised protocols derived from (average) evaluations of entire populations. To achieve a personalised approach presupposes (A) the integration of heterogeneous patients’ data with a holistic approach, to account for tumour genotypic and phenotypic heterogeneity, and (B) an omics perspective, *i.e.,* the production, via analytics methods, of a large amount of data mineable to describe and interpret tumour biology and evolution [51–54].

The NAVIGATOR biobank will allow for the collection and preservation of a large amount of high-quality, standardised imaging data and related omics data in a privacy-preserving model, including computed tomography, magnetic resonance imaging, and positron emission tomography data for various tumour settings, patients’ clinical data from regional healthcare services, and other omics data. These imaging data will be used for the extraction of imaging biomarkers based on image analysis tools for radiomics and the identification and testing of novel imaging biomarkers through big data analytics and AI.

### Digital patient model

Knowledge extraction in big data analysis is an evergreen challenge, and many techniques have been developed and tested in many application domains. Of course, in any domain, the dataset’s dimension and the number of data types considered are critical parameters: a highly heterogeneous set of data could be very difficult to interpret, not depending on the specific task. In oncology, current research is going towards merging radiomics into holomics (*i.e.,* secured access, sharing, and integration of all health data) for precision/personalised medicine: radiomics algorithms now include genomics and immunomics data to improve patient stratification [55]. For example, radiogenomics and radioimmunomics, alone or in combination with other data, improve the accuracy of the prediction of prognosis, treatment response, and outcomes (overall survival or toxicity) [56–59].

In NAVIGATOR, an initial large set of imaging and integrative omics data will be collected for three cancer types. A key issue for data collection is to have proper patient stratification. Even if unsupervised techniques have been used largely in genomics for patient stratification, such as clustering [60], deep neural networks [61, 62], and topological data analysis [63], they suffer from interpretability and could not be considered trustworthy by medical doctors. All the data collected in NAVIGATOR (*i.e.,* standard and novel imaging biomarkers, non-imaging data, and health agency data) will be integrated with prior medical knowledge (for patient stratification) and used to create a digital patient model, to support the reliable prediction of the disease phenotype and patients’ risk stratification.

Merging insights from clinical-, imaging-, and molecular-based data in the digital patient model will ensure a more comprehensive risk stratification in oncology with high accuracy, paving the way to true precision oncology. Of course, specific issues arising from the use of AI algorithms and data Analysis will be addressed, such as reproducibility, bias assessment, and the monitoring of the prediction performances.

### Data integration

In NAVIGATOR, data integration is a fundamental activity as it provides the necessary technological means to connect different and heterogeneous data sources. The purpose of the biobank of NAVIGATOR is to set up an infrastructure that can store and give access to images and biological markers for three different cancer types. Besides these kinds of datasets, the NAVIGATOR biobank also considers exploiting an additional data source, provided by Agenzia Regionale di Sanità, containing medical administrative records. These records contain information about the interaction of patients with the local health service, including their hospitalisation, prescribed drugs, and others. This data is different from the typical focused data on a specific pathology but can provide an overview of the patient's historical health life.

The NAVIGATOR project plans to evaluate the use of this data source in several aspects, including the following:

- To enable the extraction of biomarkers considering also the administrative medical information of the patient
- To help in assessing the accuracy of biomarkers by comparing their relevance with patients affected with different administrative records
- To determine the correspondence of the medical administrative history of a patient to the values of specific biomarkers

### Ethical and legal aspects

There are many questions to be addressed in the creation of a biobank, including the scale of collaboration, the need for data sharing, written rules, and regulations to cover areas of responsibility from data ownership to research dissemination [26]. The formal management structure arising from NAVIGATOR project will be realised through the following:

- A joint controllers agreement for health personal data management, reflecting the compliance with principles of lawfulness, fairness and transparency, purpose limitation, data minimisation, accuracy, storage limitation, integrity, and confidentiality
- The realisation of a dedicated broad consent defining the meaning of future research (primary use) and a systematic approach for the collecting of medical imaging and clinical data
- The adoption of a protocol for the reuse of archived personal data that will be subject to an initial and continuous assessment by the competent ethical committees and by other authorities
- The correct conservation and management of images and connected data
- The guarantee of the provision of all useful information to the person from whom the material was originally taken and the protection of the integrity of the images and personal data itself
- The respect of the duty to inform donors, families, institutions, and public and private entities about who (research groups or laboratories) collaborate with the infrastructure or about the results of the activities carried out.

## Conclusion

The general goal of the NAVIGATOR project is to advance imaging-based translational research for precision medicine in oncology, through quantitative imaging and integrative omics analyses. To this aim, NAVIGATOR will deliver an infrastructure that will offer the collection and preservation into an imaging biobank with a large amount of imaging and related omics data. To process the integrative omics data, a dedicated VRE tools will be available for the extraction of imaging biomarkers and the identification and testing of novel imaging biomarkers. The biobank will contain an initial large set of imaging and omics data (more than 1,500 patient cases), collected during the project course, for three cancer types that cover the most clinically relevant and impacting cases. For each considered cancer type will be created a digital patient model which schematises the cancer phenotyping, stratified risks, and responsivity to therapy. Finally, at the end of the project, the main issues related to the compliance with legal and ethical issues for the creation of an imaging biobank will be addressed. The achieving of this goal is essential given the lack of systematic international or national rules that address the whole of these issues.

## Data Availability

The provided information and the status of the NAVIGATOR project are attainable by the NAVIGATOR official webpage (http://navigator.med.unipi.it) and LinkedIn informative pages (https://www.linkedin.com/company/navigator-tuscany/).

## Abbreviations

AI: Artificial intelligence
EU: European Union
GDPR: General Data Protection Regulation
MRI: Magnetic resonance imaging
PACS: Picture archiving and communication system
VRE: Virtual research environment

## Footnotes

1 https://www.gcube-system.org/

2 https://www.garr.it/it/
